# Supplementary material for: Evaluation of Machine Learning Interatomic Potentials for the Properties of Gold Nanoparticles
Source: Nanomaterials (Basel). 2022 Nov 3;12(21):3891. doi: 10.3390/nano12213891 (PMC9655512; doi:10.3390/nano12213891)
Supplement: Supplementary file 1 [file nanomaterials-12-03891-s001.zip › nanomaterials-1986751-supplementary.pdf]

Supporting Information

EVALUATION OF MACHINE LEARNING INTERATOMIC POTENTIALS FOR THE PROPERTIES OF GOLD NANOPARTICLES

Marco Fronzi[1], Roger D. Amos[1], Rika Kobayashi[2], Naoki Matsumura[3], Kenta Watanabe[3] and Rafael Morizawa[3]

1 University of Technology Sydney, Ultimo, NSW 2007, Australia
2 Australian National University, Canberra, ACT 2601, Australia
3 Fujitsu Limited, Kawasaki 211-8588, Japan

All geometries are in Ångstrom.

```
Au 14.9161 14.7267 12.7538
Au 15.1770 14.6929 17.2486
Au 18.6289 17.3467 14.8573
Au 12.0813 14.7358 13.4148
Au 16.2826 17.2701 16.4651
Au 12.3833 14.5482 16.7265
Au 17.6895 14.8707 13.3864
Au 18.9054 12.7067 14.9080
Au 16.7277 12.3842 16.7387
Au 16.1357 17.2479 13.4771
Au  9.9318 14.9598 15.2912
Au 11.2835 12.4626 15.0635
Au 14.0243 12.2953 16.0379
Au 11.7081 17.1127 15.1394
Au 13.5995 17.0245 17.3083
Au 20.2994 15.1263 14.7089
Au 13.4861 12.2720 13.2154
Au 17.9549 14.9872 16.4833
Au 16.1771 12.4420 13.9393
Au 13.4061 17.1253 12.7829
```

Table S1: Initial geometry of structure I

Au	14.4351	13.6295	12.0723
Au	16.4180	15.0001	13.6927
Au	12.6681	16.5541	16.4578
Au	15.5112	13.3023	16.1731
Au	14.7016	19.1500	12.0159
Au	13.6869	11.9841	14.3985
Au	18.3067	13.4982	15.3402
Au	14.5694	14.9998	18.3054
Au	14.4350	16.3710	12.0727
Au	13.6868	18.0163	14.3990
Au	15.5111	16.6974	16.1732
Au	18.3066	16.5018	15.3405
Au	11.7799	14.9998	18.6096
Au	12.6919	14.9999	13.9164
Au	16.5633	17.8754	13.6877
Au	12.6682	13.4456	16.4578
Au	14.7017	10.8505	12.0153
Au	16.5634	12.1248	13.6873
Au	17.3016	14.9999	17.7301
Au	20.0883	14.9999	16.8773

Table 2: Initial geometry of structure II

Au	13.5932	11.8558	16.4334
Au	12.5014	15.4507	13.4474
Au	16.4069	11.8558	16.4334
Au	12.5071	12.6133	13.8479
Au	19.2354	16.5745	15.6193
Au	10.1611	14.0513	14.4012
Au	12.6009	18.1964	14.2254
Au	16.4355	16.7505	16.5991
Au	15.0000	16.8584	13.4035
Au	15.0000	19.0054	15.4777
Au	13.5646	16.7504	16.5988
Au	19.8389	14.0513	14.4012
Au	12.1761	14.2519	16.4562
Au	15.0000	14.0267	13.0857
Au	17.4928	12.6133	13.8479
Au	17.8238	14.2519	16.4561
Au	17.3991	18.1963	14.2255
Au	15.0000	14.3014	17.0572
Au	17.4986	15.4507	13.4472
Au	10.7646	16.5745	15.6193

Table S3: Initial geometry of structure III

Au 14.93345 14.72763 12.80748
Au 15.16630 14.73349 17.22263
Au 18.42439 17.19627 14.85994
Au 12.24798 14.75144 13.41856
Au 16.19623 17.19519 16.42114
Au 12.50141 14.62596 16.68746
Au 17.58510 14.83717 13.42134
Au 18.68341 12.75021 14.91917
Au 16.67211 12.46421 16.77643
Au 16.03039 17.13686 13.58993
Au 10.08721 14.91657 15.25070
Au 11.53723 12.61859 15.04716
Au 14.10567 12.43444 15.98036
Au 11.90931 16.95712 15.13248
Au 13.64840 17.05774 17.21209
Au 20.10871 15.04523 14.70236
Au 13.56103 12.31107 13.19218
Au 17.81810 14.96396 16.44177
Au 16.12193 12.49458 13.98546
Au 13.46007 17.11992 12.87767

Table S4: Geometry of structure I optimised by VASP
E=-48.7914 ev.

Au 14.56245 13.67519 12.03008
Au 16.51864 15.00362 13.54243
Au 12.71151 16.46661 16.50299
Au 15.47574 13.39918 16.05933
Au 14.62394 19.04301 12.20449
Au 13.58277 12.19908 14.36592
Au 18.16390 13.53233 15.27120
Au 14.58280 14.98080 18.20110
Au 14.56385 16.34640 12.03463
Au 13.60773 17.80935 14.37873
Au 15.47981 16.58305 16.06590
Au 18.17593 16.45946 15.28096
Au 11.90952 15.00274 18.62361
Au 13.45081 15.00526 14.22945
Au 16.47101 17.80401 13.73208
Au 12.70383 13.53634 16.49932
Au 14.59298 10.97393 12.18989
Au 16.45265 12.20396 13.71417
Au 17.18514 14.99434 17.62186
Au 19.77987 14.98157 16.87466

Table S5: Geometry of structure II optimised by VASP
E=-50.6370 ev.

Au	13.67095	11.92837	16.43126
Au	12.64137	15.43857	13.47449
Au	16.35374	11.93730	16.47103
Au	12.73572	12.68644	13.96545
Au	19.05806	16.50018	15.51933
Au	10.37558	14.11164	14.34794
Au	12.72875	18.14618	14.23728
Au	16.37631	16.70050	16.54147
Au	14.99759	16.80987	13.48658
Au	14.99944	18.91247	15.58880
Au	13.61639	16.69299	16.53385
Au	19.61879	14.11918	14.33925
Au	12.31536	14.26637	16.36942
Au	14.99793	14.05021	13.23102
Au	17.25401	12.69284	13.97593
Au	17.69004	14.27883	16.37128
Au	17.27001	18.14628	14.23676
Au	15.00164	14.32766	16.97119
Au	17.35528	15.44202	13.47186
Au	10.94304	16.49278	15.51912

Table S6: Geometry of structure III optimised by VASP
E=-48.8983

Au	14.8990	14.5895	12.5354
Au	15.2071	14.5688	17.5146
Au	18.0153	17.2783	14.8740
Au	12.2072	14.6983	13.1346
Au	15.7588	17.0277	16.3482
Au	12.5335	14.4509	17.0426
Au	17.4592	14.9302	13.3717
Au	18.6476	12.8699	14.8747
Au	16.6960	12.4374	16.7236
Au	15.6415	17.0361	13.6151
Au	10.7498	15.5526	15.2725
Au	11.5413	12.8413	15.0693
Au	14.1035	12.4521	15.9933
Au	13.2429	16.7193	15.0566
Au	13.5452	16.8149	17.8370
Au	19.8770	15.2773	14.7540
Au	13.5128	12.2717	13.2277
Au	17.7133	15.0263	16.5124
Au	16.0880	12.5635	13.9557
Au	13.3593	16.9315	12.2334

Table S7: Geometry of structure I optimised by LAMMPS
E=-51.0285 ev.

Au	14.5835	13.6737	12.0331
Au	16.5625	15.0001	13.5079
Au	12.7060	16.4637	16.4869
Au	15.4753	13.3839	16.0870
Au	14.6197	19.0157	12.1983
Au	13.5986	12.2096	14.3693
Au	18.1565	13.5362	15.2876
Au	14.5805	14.9998	18.1941
Au	14.5834	16.3268	12.0333
Au	13.5985	17.7904	14.3697
Au	15.4752	16.6160	16.0873
Au	18.1565	16.4639	15.2878
Au	11.9180	14.9997	18.6070
Au	13.4059	15.0000	14.2023
Au	16.4579	17.7905	13.7407
Au	12.7060	13.5360	16.4866
Au	14.6198	10.9847	12.1977
Au	16.4579	12.2097	13.7403
Au	17.1716	14.9999	17.6241
Au	19.7616	14.9999	16.8815

Table S8: Geometry of structure II optimised by LAMMPS
E=-52.0229 ev.

Au	13.6963	11.9221	16.4258
Au	12.6302	15.4836	13.3099
Au	16.3664	11.9101	16.5019
Au	12.8520	12.7770	13.9819
Au	18.9193	16.4900	15.5362
Au	10.4770	14.0948	14.3998
Au	12.7928	18.0951	14.2333
Au	16.3487	16.6624	16.6253
Au	14.9946	16.8756	13.2025
Au	14.9970	18.8400	15.5807
Au	13.6523	16.6546	16.6267
Au	19.5077	14.0911	14.3981
Au	12.3583	14.2473	16.4516
Au	14.9924	14.1403	13.1256
Au	17.1192	12.7800	14.0225
Au	17.6559	14.2623	16.4742
Au	17.1995	18.0972	14.2264
Au	15.0052	14.2951	17.0995
Au	17.3562	15.4780	13.3132
Au	11.0787	16.4841	15.5484

Table S9: Geometry of structure III optimised by LAMMPS
E=-51.3517 ev.

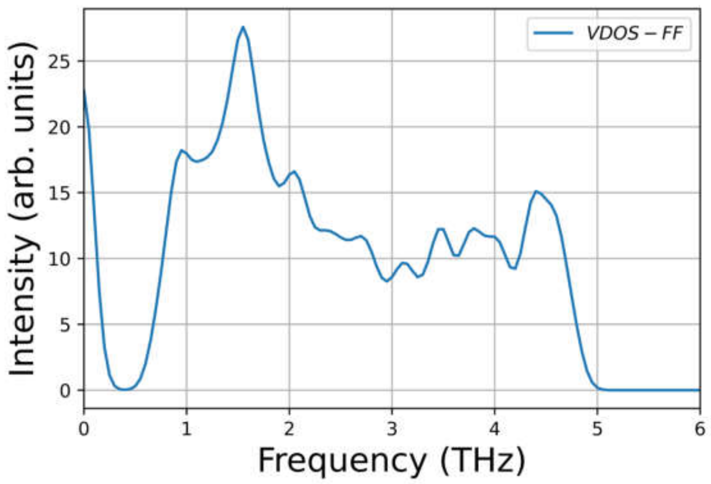

Figure S10: Phonon Density of States of structure II computed within the frozen phonon approximation, for a (1x1x1) expansion of the supercell**.**

Example of the training parameters used in the DeePMD fitting of the potential.

```
{
  "_comment": " model parameters",
    "model": {
        "type_map":     ["Au"],
        "descriptor" :{
            "type": "se_a",
            "sel":              [20],
            "rcut_smth":        0.58,
            "rcut":             6.00,
            "neuron":           [40,80,160],
            "resnet_dt":        false,
            "axis_neuron":      16,
            "seed":             1,
            "_comment":         " that's all"
        },
        "fitting_net" : {
            "neuron":           [200, 200, 200],
            "resnet_dt":        true,
            "seed":             1,
            "_comment":         " that's all"
        },
        "_comment":     " that's all"
    },

    "learning_rate" :{
        "type":         "exp",
        "decay_steps":  5000,
        "start_lr":     0.001,
        "stop_lr":      1.0e-8 ,
        "_comment":     "that's all"
    },

    "loss" :{
        "type":         "ener",
        "start_pref_e": 0.02,
        "limit_pref_e": 1,
        "start_pref_f": 1000,
        "limit_pref_f": 1,
        "start_pref_v": 0,
        "limit_pref_v": 0,
        "_comment":     " that's all"
    },

    "training" : {
        "training_data": {
            "systems":          ["Au20-all-training"],
            "batch_size":       "auto",
            "_comment":         "that's all"
        },
        "validation_data":{
            "systems":          ["Au20-all-validation"],
            "batch_size":       1,
            "numb_btch":        3,
            "_comment":         "that's all"
        },
        "numb_steps":   500000,
        "seed":         1,
        "disp_file":    "lcurve.all..out",
        "disp_freq":    1000,
        "save_freq":    1000,
        "_comment":     "that's all"
    },

    "_comment":         "that's all"
```
